# Inhibition Potencies of Phytochemicals Derived from Sesame Against SARS-CoV-2 Main Protease: A Molecular Docking and Simulation Study

**DOI:** 10.3389/fchem.2021.744376

**Published:** 2021-10-08

**Authors:** Anuj Kumar, Dwijesh Chandra Mishra, Ulavappa Basavanneppa Angadi, Rashmi Yadav, Anil Rai, Dinesh Kumar

**Affiliations:** ^1^ Centre for Agricultural Bioinformatics (CABin), ICAR- Indian Agricultural Statistics Research Institute, New Delhi, India; ^2^ Division of Germplasm Evaluation, ICAR-National Bureau of Plant Genetic Resources, New Delhi, India

**Keywords:** COVID-19, main protease, sesame, natural compounds, molecular docking, molecular dynamics simulations, therapeutics

## Abstract

The ongoing COVID-19 pandemic, caused by SARS-CoV-2, has now spread across the nations with high mortality rates and multifaceted impact on human life. The proper treatment methods to overcome this contagious disease are still limited. The main protease enzyme (M^pro^, also called 3CL^pro^) is essential for viral replication and has been considered as one of the potent drug targets for treating COVID-19. In this study, virtual screening was performed to find out the molecular interactions between 36 natural compounds derived from sesame and the M^pro^ of COVID-19. Four natural metabolites, namely, sesamin, sesaminol, sesamolin, and sesamolinol have been ranked as the top interacting molecules to M^pro^ based on the affinity of molecular docking. Moreover, stability of these four sesame-specific natural compounds has also been evaluated using molecular dynamics (MD) simulations for 200 nanoseconds. The molecular dynamics simulations and free energy calculations revealed that these compounds have stable and favorable energies, causing strong binding with M^pro^. These screened natural metabolites also meet the essential conditions for drug likeness such as absorption, distribution, metabolism, and excretion (ADME) properties as well as Lipinski’s rule of five. Our finding suggests that these screened natural compounds may be evolved as promising therapeutics against COVID-19.

## Introduction

The ongoing pandemic eruption due to the worldwide spread of coronavirus disease (COVID-19) is caused by the novel virus strain severe acute respiratory syndrome coronavirus-2 (SARS-CoV-2; previously named 2019-nCoV) (Wu et al., 2020b). This viral disease is an unprecedented global public health care threat (Jamwal et al., 2020). The first case of COVID-19 disease was originated from Wuhan, Hubei Province, China, and quickly spread across 219 countries and territories around the world with high mortality rates in immunocompromised patients (Enayatkhani et al., 2020; Mackenzie and Smith, 2020; Xu et al., 2020). Based on the recommendations of the Emergency Committee, the World Health Organization (WHO) has declared this respiratory infectious disease as a Public Health Emergency of International Concern (PHEIC) on 30 January, 2020 and a pandemic on 11 March, 2020 (Shi et al., 2020; Yu et al., 2020). As on 10 July 2021, this contagious disease had led to more than 185,291,530 confirmed cases and 4,010,834 fatalities (https://covid19.who.int/), with the number of cases increasing abruptly across the globe. At present, India is fighting hard against the second wave of COVID-19. The ongoing pandemic has now initiated taking a toll on India’s economy. A large population of India is facing disproportionately higher rates of COVID-19 infection, morbidity, and mortality. As of 10 July 2021, the total COVID-19 caseload has now soared to 30,752,950 with 405,939 deaths (https://covid19.who.int/table). India is the most severely affected Asian country. The ongoing pandemic has been considered more dreadful than the previous global outbreaks, namely, SARS-CoV (2002–2003) and Middle East respiratory syndrome (MERS) (2012–present) (de Wit et al., 2016; Gupta et al., 2020; Wang et al., 2020b; Wu and McGoogan, 2020; Yuan et al., 2020). Based on previous investigations, the fatality rate of SARS-CoV and MERS has been calculated as 10 and 35%, respectively (Lee et al., 2004; Cheng et al., 2007). It has been well-reported that COVID-19 affects the lower respiratory tract of the body, which causes pneumonia and affects the gastrointestinal system, kidney, heart, and central nervous system. Fever, cough, diarrhea, and tiredness have been considered the most common symptoms (Chen et al., 2020a; Tang et al., 2020), while aches and pains, sore throat, conjunctivitis, headache, loss of taste or smell, a rash on skin, or discoloration of fingers or toes are the less common symptoms of this infectious disease (Backer et al., 2020; Rothe et al., 2020; Russell et al., 2020; Verdoni et al., 2020; Yu and Yu, 2020).

The coronaviruses have been recognized as a large enveloped positive-sense single-strand RNA viruses from Nidovirales (order) of the Coronaviridae family and subfamily Coronavirinae (Raj et al., 2021; Shamsi et al., 2021). This subfamily is classified into four genera including *alpha-*, *beta-*, *gamma-*, and *deltacoronavirus* (*α-*, *β-*, *γ-*, and *δ-CoV*) based on evolutionary methods (Hulswit et al., 2016). In view of previous reports, coronaviruses have been considered as highly evolving viruses, with a high rate of mutation and genomic recombination (Chen et al., 2020b). In the past, six species of human coronavirus associated with different respiratory tract diseases have been reported, which include HCoV-NL63, HCoV-229E, HCoV-OC34, HCoV-HKU1, SARS-CoV, and MERS-CoV (Arden et al., 2005; Woo et al., 2005; Su et al., 2016). The novel strain SARS-CoV-2 has been characterized as the seventh strain of the human coronavirus. Based on the significant nucleotide sequence similarity with SARS and MERS coronaviruses, the International Committee on Taxonomy of Viruses (ICTV) coined the nomenclature of SARS-COV-2 (Hasan et al., 2020). The ICTV taxonomically placed the SARS-COV-2 in the genus *Betacoronavirus* (Helmy et al., 2020; Wang et al., 2020b).

The genome size of SARS-CoV-2 is ∼29.9 kb (29,903 nucleotides) (Wu et al., 2020a). The first whole-genome sequencing data for SARS-CoV-2 (∼30 kb) were submitted to the Genbank with the accession number MN908947 and isolated from Wuhan (Wu et al., 2020a). The genome of SARS-CoV-2 encodes approximately 13–15 open reading frames (ORFs) which are flanked by 5′ and 3′ UTRs (Chen et al., 2020b; Elfiky and Azzam, 2020; Gordon et al., 2020). These ORFs constitute a replicase assembly during the replication process of the central dogma of molecular biology and encode 27 distinct structural and non-structural proteins (NSPs) (Liu et al., 2021; Shamsi et al., 2021). The 5′ end of the SARS-CoV-2 genome encodes 16 NSPs (Nsp1-16) and constitutes the replicase/transcriptase complex (RTC). These 16 proteins are conserved in all SARS viruses and play a critical role in a set of biological processes such as viral replication, assembly, and immune response modulation (Shamsi et al., 2021). The 3′ end of the viral genome encodes four conical structural proteins including E (envelope protein), M (membrane protein), N (nucleocapsid protein), and S (spike protein), and nine putative accessory factors. The main protease enzyme (M^pro^ also called 3CL^pro^) is essential for viral replication and has been considered as one of the potent drug targets for treating COVID-19 (Joshi et al., 2020; Khan et al., 2020; Kumar et al., 2020; Pant et al., 2020; Wu et al., 2020a; Zhang et al., 2020). In cooperation with other components, this important enzyme also helps in the transcription of the viral RNA. M^pro^ is a key enzyme that exclusively cleaves the polyproteins (pp1a and pp1ab) which is essential for the assembly of virus drugs (Jin et al., 2020). The molecular mass of M^pro^ is 33,797 Da with length of 306 amino acid residues and structurally possesses the three functional domains, namely, domain I (8–101 residues), domain II (102–184 residues), and domain III (201–306 residues) (Jin et al., 2020; Khan et al., 2020). Among them, domains I and II have an antiparallel β-barrel structure, while domain III represents a group of five α-helices organized as a large antiparallel cluster. Domain III is connected to domain II by a 15-residue-long loop region (185–200 residues). The active site is composed of a catalytic dyad having Cys145 and His41 residues (Khan et al., 2020). The functional role of M^pro^ in the viral replication highlights its importance that can be used to identify the potential drug therapeutics against COVID-19 (Ullrich and Nitsche, 2020). Solved crystal structures of M^pro^ provide a platform to develop and design the antiviral drugs to combat COVID-19 (Jin et al., 2020; Zhang et al., 2020). In response to the COVID-19 outbreak, several studies have been performed using integrated bioinformatics and molecular modeling approaches for the screening of novel natural metabolites as potential drug targets against M^pro^ (Chikhale et al., 2020a; Kumar et al., 2020; Maurya and Sharma, 2020; Rout et al., 2020; Tripathi et al., 2020; Mishra et al., 2021; Romeo et al., 2021; Tock et al., 2021). But no effective method has been developed yet to prevent and treat the COVID-19 disease in a significant manner. In addition to the aforementioned approaches, several other viral protease inhibitors like remdesivir, hydroxychloroquine, chloroquine, lopinavir, ritonavir, oseltamivir, and fapilavir have been explored as repurposed drugs for COVID-19 treatment (Chang et al., 2016; Chang et al., 2020; Contini, 2020; Das et al., 2020; Elfiky, 2020; Gonzalez–Paz et al., 2020; Islam et al., 2020; Khan et al., 2020; Sinha et al., 2020; Wahedi et al., 2020; Abdelli et al., 2021). The antimalarial drug named as chloroquine has been proposed as the potential inhibitor of M^pro^ activity (Ou et al., 2021). In a recent follow-up study, Pathak et al. (2021) explored the potential of rifampicin and letermovir as repurposed drug candidates against COVID-19. On the contrary, several studies reported the severe adverse effects of these repurposed drugs in different countries (Sultana et al., 2020; Wang et al., 2020a). Therefore, it is imperative to discover natural compound–based drug targets that could serve as potential inhibitors of different SARS-CoV-2 proteins and aid in controlling viral replication to enhance efficacy in COVID-19 treatment.

Sesame (*Sesamum indicum* L.) is an herbaceous annual plant cultivated for its edible seed, oil, and flavorsome value, belonging to the order Tubiflorae, family Pedaliaceae with many common names including gingelly, til, and benne seed (Bhat et al., 2014; Pathak et al., 2019). This oil crop is regarded as “queen of oilseeds” because of its property of resistance to oxidation and rancidity (Dalibalta et al., 2020). Sesame is widely cultivated in the tropical parts of Africa and Asia, India being one of the major producers with Myanmar, China, and Sudan (Majdalawieh et al., 2017). A plethora of nutrients including proteins, carbohydrates, antioxidants, lignans, tocopherols, phytates, phytosterols, and polyunsaturated fatty acids are exclusively found in sesame (Nagendra Prasad et al., 2012; Kumar et al., 2018; Pathak et al., 2019). These bioactive compounds possess certain medicinal properties like hepatoprotective, hypoglycemic, antihypertensive, anti-estrogenic, and anticancer (Kumar and Singh, 2014; Majdalawieh et al., 2017). Active ingredients of sesame have also been investigated as potential inhibitors of Parkinson’s disease (PD) (Kappo et al., 2016). There are very few reports available for the screening of sesame-derived compounds against main protease of COVID-19. So far, only one compound of sesame, namely, sesamin has been well-explored against COVID-19 using *in silico* approach. Kodchakorn et al. (2020) investigated the potential of sesamin along with other herbal medicines (andrographolide, anthocyanin-b-D-glucoside, capsaicin, curcumin, cyanidin, cyanidin-3-O-glucoside, and hesperidin) against the M^pro^ of SARS-CoV-2 using molecular docking. Docking complexes of these nutraceuticals with M^pro^ were further validated for their atomic stability using molecular dynamics (MD) simulations on 50 ns, and suggested that the screened compounds may be considered for coprotection and treatment against COVID-19. In a recent study, Pandey and Verma (2020) also studied the potential of sesamin and four other dietary components (galangin, ellagic acid, capsaicin, and epicatechin) as structural inhibitors of SARS-CoV-2 M^pro^ using the molecular docking approach. In a very recent study, Allam et al. (2021) reported seven sesame-derived natural compounds (sesamin, sesamolin, pinoresinol, hydroxymatairesinol, spicatolignan, ferulic acid, and vanillic acid) as potential inhibitors against three proteins of SARS-CoV-2 including M^pro^, papain-like protease (PL^pro^), and the RNA-dependent RNA polymerase (RdRp) using the molecular docking analysis followed by MD simulations on 50 ns for representative complexes. However, there is no significant evidence of docking results evaluation available for MD simulations on high nanosecond scale (up to 200 ns) to understand the inhibitory mechanism of all sesame-derived compounds against the SARS-CoV-2 proteins. Despite the medicinal importance of sesame, all bioactive molecules derived from this important medicinal plant have not been well-explored in a significant manner yet for the treatment of COVID-19. With the fruitful utilization of molecular modeling methods including molecular docking and MD simulations, sesame-derived bioactive compounds may be utilized to design the alternative natural compound–based effective therapeutics against COVID-19.

Keeping this in view, in the present study, we have undertaken a thorough attempt to investigate the inhibition potencies of 36 phytochemicals from sesame against M^pro^ of SARS-CoV-2 using the molecular docking approach. Four natural metabolites, namely, sesamin, sesaminol, sesamolin, and sesamolinol, were further subjected to conformational stability using MD simulations followed by free energy calculations. The knowledge generated in the current study encourages and suggests that the sesame-derived phytochemicals have enough potential of being effective in treatment of COVID-19.

## Materials and Methods

**FIGURE 1:**
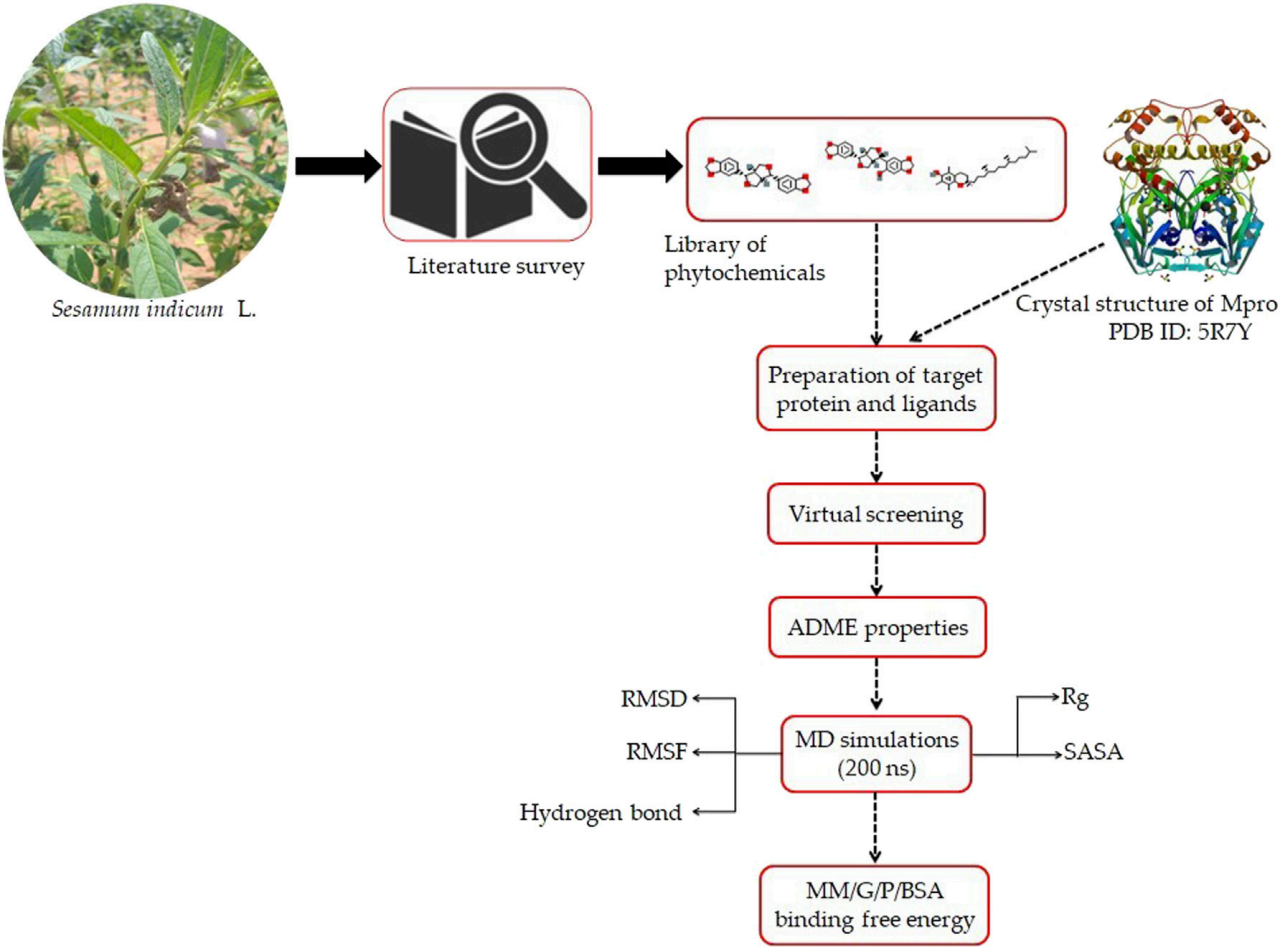
Representation of pipeline utilized in the present study to identify the inhibitors of M^pro^ of SARS-CoV-2 using an extensive molecular modeling approach.

A flowchart depicting the pipeline involved in the identification of interaction between sesame-derived bioactive molecules and M^pro^ is presented in Figure 1.

### Ligand Selection

An extensive literature survey was conducted to prepare a library of sesame-derived natural compounds reported with therapeutic potential. Chemical structures of 36 phytochemicals (Supplementary Table S1) were obtained from the PubChem database (Kim et al., 2020) in a Spatial Data File (SDF) format. All these molecules were optimized prior to molecular docking using a set of AutoDock tools (Morris et al., 2009). Each and every molecule embedded in thse library was prepared with the addition of polar hydrogens and Gasteiger charges calculation. For the docking purpose, the molecules were saved in a pdbqt format using PyRx Open Babble tools (O'Boyle et al., 2011).

### Preparation of Receptor

The crystal structure of the M^pro^ of SARS-CoV-2 in a complex with Z45617795 (PDB ID: 5R7Y) was attained from the RCSB-Protein Data Bank (Berman, 2000; Burley et al., 2018) for docking purposes. This protein crystal structure was solved by the PanDDA analysis group (https://www.rcsb.org/structure/5R7Y). Preprocessing of the M^pro^ of SARS-CoV-2 was carried out by removing water atoms and heteroatoms, and adding polar hydrogen atoms and Kollman charges on it using AutoDockTools version 1.5.6. Swiss-pdb Viewer (Guex and Peitsch, 1997) was employed to structure optimization and energy minimization. The clean geometry module available in the Discovery Studio platform was utilized for the side chain correction.

### Virtual Screening Based on Molecular Docking

In a search for a drug against COVID-19, we performed a site-specific docking screen for the M^pro^ of SARS-CoV-2 against the prepared library of sesame-derived natural compounds containing 36 compounds. AutoDock Vina program was employed for virtual screening. The grid box was created with the size of 70 Å × 70 Å × 70 Å, with a total of 50 genetic run. For the purpose of docking, amino acid residues such as Thr24, Thr26, Asn119, Phe140, Gly143, Cys145, His163, His164, Glu166, Gln189, and Thr190 were considered as active sites, as earlier reported by Khan et al. (2020) and Kumar et al. (2020). Other parameters were set as default while docking process. The carmofur (CID_2577) compound was selected as the positive control (Jin et al., 2020) for docking process. After docking, the top ranked compounds (based on docking score, number of hydrogen bonds, and specificity) (Table1) were chosen and visually inspected using PyMol and Discovery Studio (DeLano, 2002).

**TABLE 1:** List of top four natural compounds shortlisted based on binding energy score as a result of virtual screening.

S. No.	Compound	2D structure	Binding energy (kcal/mol)	Molecular interactions
1	M^pro^ (active site residues)	Thr24, Thr26, Asn119, Phe140, Gly143, Cys145, His163, His164, Glu166, Gln189, and Thr190		
2	Sesamin (CID_72307)	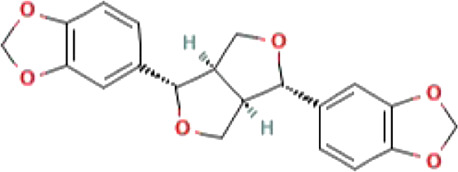	−6.7	Hydrogen bond: ASN151 (5.46 Å), SER158 (4.38 Å), and ARG298 (6.05 Å)
				Carbon–hydrogen bond: ASP295 (5.38 Å)
				Alkyl: VAL104 (5.27 Å)
				Pi–sigma: VAL104 (4.29 Å)
3	Sesaminol (CID_94672)	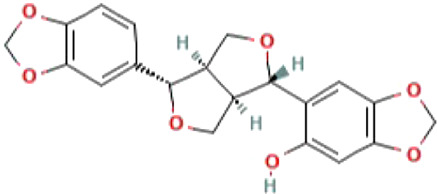	−6.6	Hydrogen bond: ARG105 (6.59 Å), ASN151 (5.39 Å), and ARG298 (6.05 Å)
				Carbon–hydrogen bond: ASP295 (5.27 Å)
				Pi-Sigma: VAL104 (4.30 Å)
4	Sesamolin (CID_131801617)	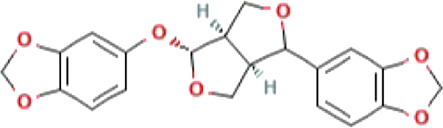	−6.4	Hydrogen bond: ARG105 (6.03 Å), GLN110 (4.52 Å), and SER158 (4.08 Å)
				Pi–sigma: VAL104 (4.89 Å)
5	Sesamolinol (CID_443019)	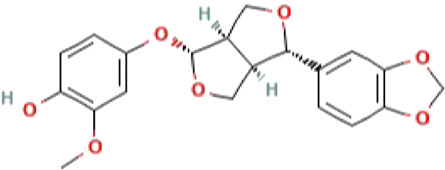	−6.1	Hydrogen bond: SER158 (4.10 Å)
				Carbon–hydrogen bond: ILE106 (4.45 Å), and GLN110 (5.21 Å)
				Pi–sigma: VAL104 (5.04 Å)
				Alkyl: VAL202 (5.45 Å), and ILE249 (5.21 Å)
				Pi-Alkyl: HIS246 (5.26 Å)

### Drug-Likeness and Absorption, Distribution, Metabolism, and Excretion Profiling

The automated Swiss ADME server (Daina et al., 2017) was employed to calculate the drug-likeness attributes of screened molecules. Different molecular properties such as molecular weight, number of hydrogen bond accepters, number of hydrogen bond donors, number of rotatable bonds, molar refractivity, bioavailability score, synthetic accessibility, TPSA, and solubility were calculated with utilizing Lipinski’s rule of five (Lipinski, 2004) and Veber’s rule (Veber et al., 2002).

### Molecular Dynamics Simulations

In order to assess the stabilities of docking conformation complexes of the four bioactive compounds sesamin, sesaminol, sesamolin, and sesamolinol with SARS-CoV-2 M^pro^, MD simulations were performed using GROMOS9643a1 force field embedded in GROMACS 5.1.1 package installed on Linux-based workstation (Abraham et al., 2015; Kutzner et al., 2019). For the MD simulations, we followed the protocol previously described by Gajula et al. (2016) and Jee et al. (2017). The automatic PRODRG server was employed to prepare the topology files of ligand molecules (Schüttelkopf and van Aalten, 2004). The docking complexes were solvated in a dodecahedron box. In order to make the whole system neutral, the appropriate Na+ ions were added to the system. The steepest descent algorithm was applied to perform the energy minimization of the prepared system with 50,000 iteration steps and cutoff up to 1,000 kjmol^−1^ with a primary goal of reducing the steric clashes during simulations. The long-range electrostatic interactions were calculated by using particle mesh Ewald (PME) truncation method (Abraham and Gready, 2011). Prior to a production run, the process of equilibrium was completed in two phases. In the first phase, equilibration was established with a constant number of particles, volume, and temperature (NVT), with each step 2 femtosecond (fs). The second phase was performed with a constant number of particles, pressure, and temperature NPT, with the ensemble at 300 K. After determining the coordinates, LINCS algorithm was considered to constrain the covalent bonds involving hydrogen atoms (Hess et al., 1997; Hess, 2007). Temperature was regulated inside the box using V-rescale, a popular Berendsen temperature coupling method. Finally, a production run of 200 ns was run with each step of 2 fs.

### Trajectory Analysis

After the successful completion of MD simulations, trajectories were analyzed using a set of tools implemented in GROMACS package. The gRMS tool of GROMACS was utilized to calculate the root-mean-square deviation (RMSD) variation in protein backbone, while the overall root-mean-square fluctuation (RMSF) in the atomic positions of protein C backbone was generated by using the grmsf module. The gyrate, gmxsasa, and g h bond tools were employed to estimate the radius of gyration (Rg), solvent accessible surface area (SASA), and hydrogen bonds, respectively.

### Molecular Mechanic/Poisson–Boltzmann Surface Area Binding Free Energy Calculations

The Molecular Mechanic/Poisson–Boltzmann Surface Area (MM/PBSA) was performed on g mmpbsa script program to calculate the binding free energy of interactions between the docking complexes (Kumari et al., 2014; Aldeghi et al., 2017). After the simulation of docking complexes, all the trajectories of 200 ns were used for MM/PBSA-based binding free energy analysis. The major energy components such as binding energy (kJ/mol), van der Waals energy (DEvdW), electrostatic energy, polar solvation energy, and SASA energy all together contributed to calculate the MM/PBSA relative binding affinity. The MM/PBSA method–based binding free energy of the protein–ligand systems were calculated using the following equation:

$$\Delta {\text{G}}_{\text{MMPBSA}}\;={\langle {\text{G}}_{\text{complex}}\;-\;{\text{G}}_{\text{protein}}\;-\;{\text{G}}_{\text{ligand}}\rangle }_{\text{complex}},$$

where *G*_*complex*_ represents the total free energy of the docking complex, and *G*_*protein*_ and *G*_*ligand*_ depict the total free energies of the isolated protein and ligand in the solvent, respectively.

## Results and Discussion

### Molecular Docking

Molecular docking is one of the most applied methods in the process of computer-aided drug design (CADD) to identify potential inhibitors against various pathogens. With this revolutionary method, an immense amount of energy, time, and costs of the drug discovery process can be saved to screen the large drug libraries for the discovery of potential drug compounds (Wadood et al., 2013; Yu and MacKerell, 2016). There is no effective cure for COVID-19 so far; therefore, identification of potential drug compounds is required on an urgent basis. In the present study, we screened an in-house library of sesame-derived bioactive molecules against M^pro^ of SARS-CoV-2 using a molecular docking approach. In total, 36 natural compounds (Supplementary Table S1) were docked into the binding pocket of M^pro^. The docking results demonstrated that out of 36 selected natural compounds used in the present study, four bioactive molecules, namely, sesamin, sesaminol, sesamolin, and sesamolinol were found to have a higher binding energy of −6.7, −6.6, −6.4, and −6.1 kcal/mol^−1^, respectively, than the positive control compound carmofur whose binding energy was determined to be −5.2 kcal/mol^−1^. These four natural compounds (sesamin, sesaminol, sesamolin, and sesamolinol) ranked as top interacting with M^pro^ based on the affinity of molecular docking, number of hydrogen bonds and compound specificity.

**FIGURE 2:**
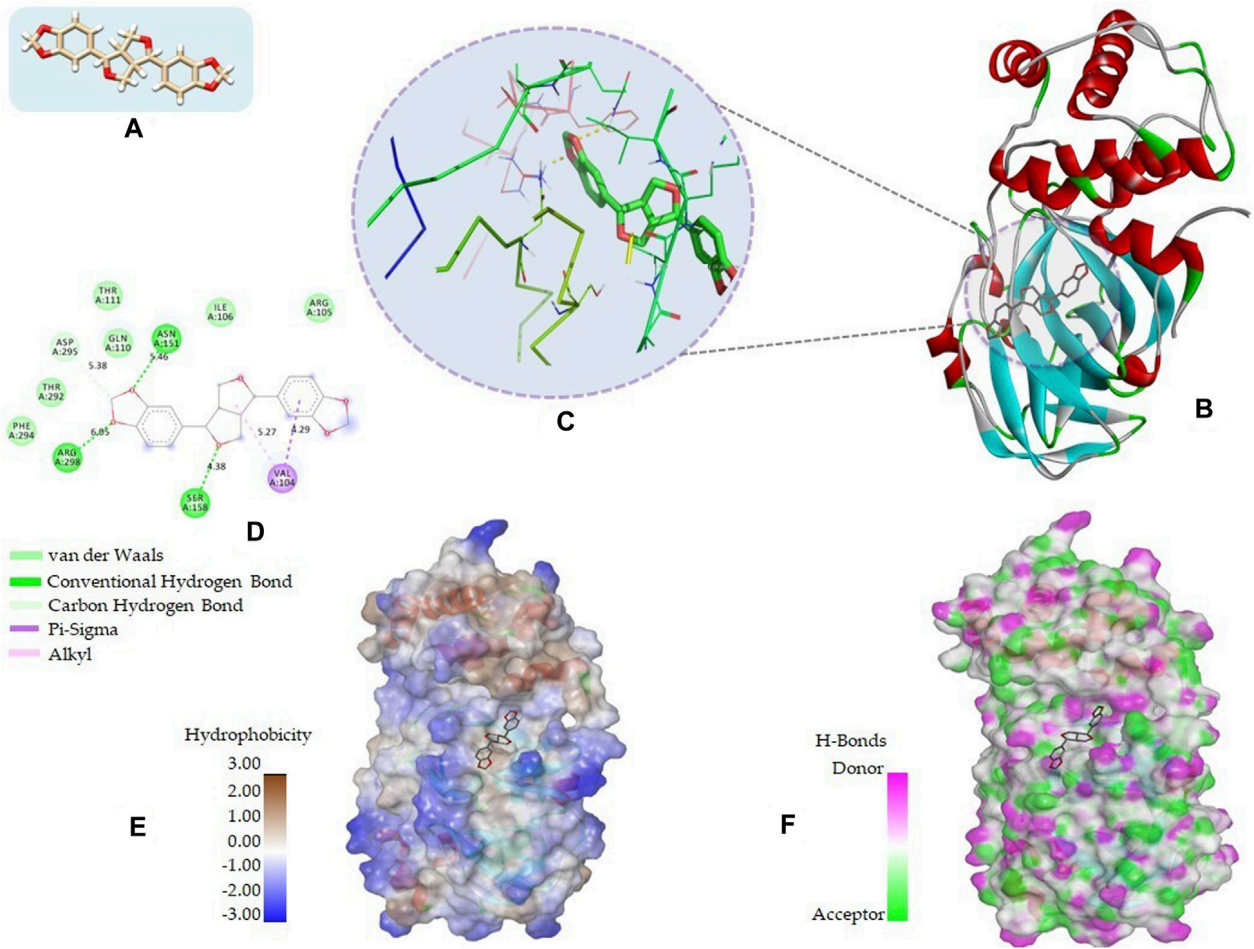
2D and 3D representation of molecular interaction between the M^pro^ of COVID-19 (PDB ID: 5R7Y) and sesamin compound (CID_72307): **(A)** 3D structure presentation of sesamin; **(B)** Molecular docking complex of a crystal structure of M^pro^ with sesamin molecule, docked using AutoDock implemented in PyRx package; **(C)** close view of pocket with sesamin structure in the stick model colored by atom types, yellow dashed lines represent the hydrogen bond networks; **(D)** 2D representation of different types of interactions with sesamin including van der Waals, conventional hydrogen bond, carbon hydrogen bond, Pi–sigma, and alkyl; **(E)** hydrophobicity surface representation of the overall structure of Mpro in complex with Sesamin; and **(F)** pocket view of sesamin binding with M^pro^ and the representation of residues involved in hydrogen bond donor acceptor. The docking complex of M^pro^ with sesamin was rendered in different CPK using UCSF Chimera, Discovery Studio, and PyMol.

The 2D structures, binding score, and details of interactions of the top four screened compounds are displayed in Table 1. Docking complexes of these natural metabolites with M^pro^ have been considered for further evaluation using MD simulations and MM/PBSA energy calculations. Discovery studio and PyMOL programs were employed to prepare the two- and three-dimensional plots of molecular interaction networks, respectively. After visualizing the 2D and 3D interaction plots, it was observed that the sesamin compound formed hydrogen bonds with three residues, namely, Asn151 (5.46 Å), Ser158 (4.38 Å), and Arg298 (6.05 Å). This compound was found to have one carbon–hydrogen (C–H) bond with Asp295 (5.38 Å), alkyl bond with Val104 (5.27 Å), and Pi–sigma bond with Val104 (4.29 Å) residue. It also manifests van der Waals (VdW) interaction with six residues including Arg105, Ile106, Gln110, Thr111, Thr292, and Phe294 (Figure 2).

**FIGURE 3:**
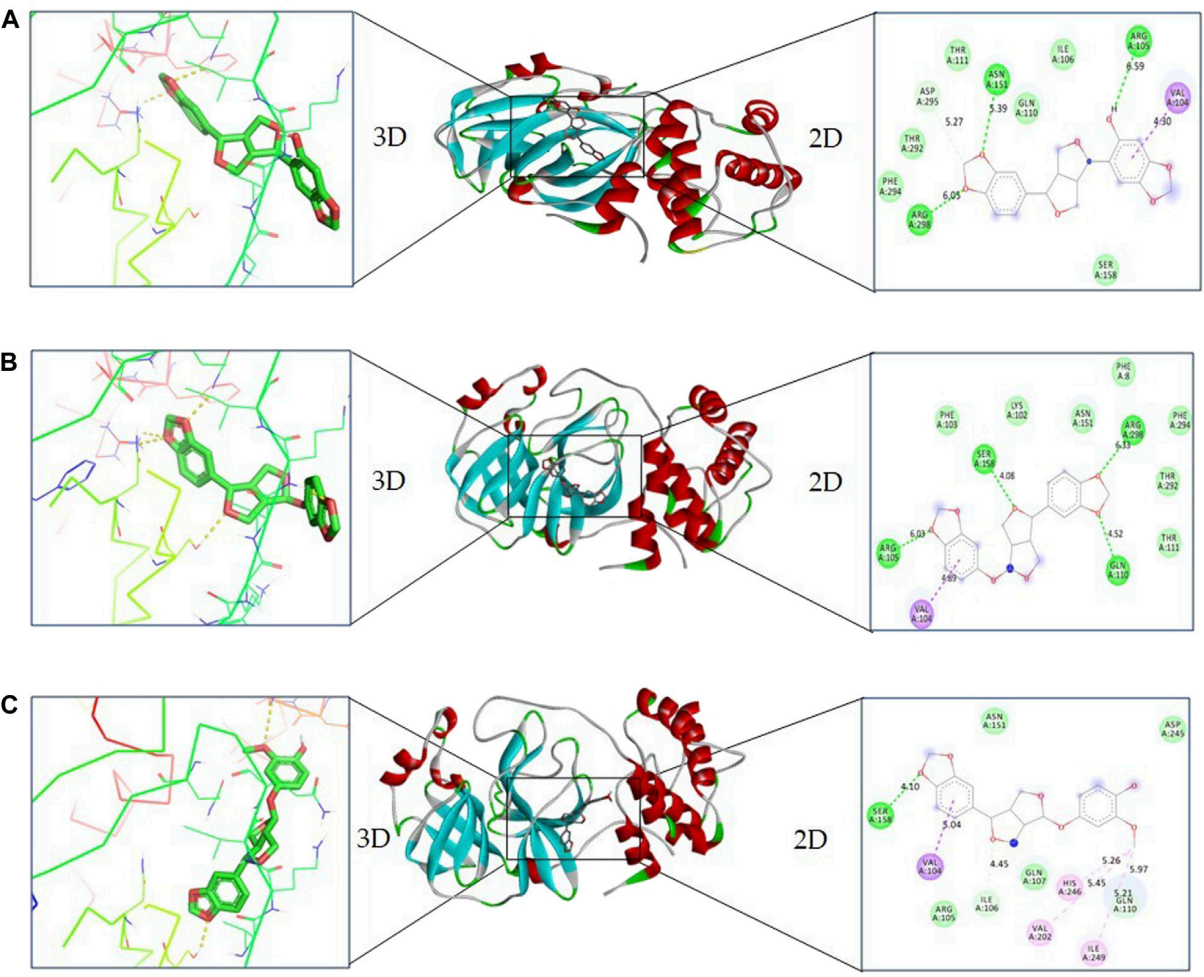
2D and 3D representation of docking complexes: **(A)** M^pro^ and sesaminol complex; **(B)** M^pro^ and sesamolin complex; **(C)** M^pro^ and sesamolinol complex visualized using UCSF Chimera and Discovery Studio.

In the case of sesaminol, three residues, namely, Arg105 (6.59 Å), Asn151 (5.39 Å), and Arg298 (6.05 Å), formed the hydrogen bonds. Residues Asp295 (5.27 Å) and Val104 (4.30 Å) interacted via C–H bond and Pi–sigma, respectively. Five residues including Ile106, Gln110, Thr111, Thr292, and Phe294 manifest VdW interaction (Figure 3A). As shown in Figure 3B, sesamolin molecule exhibits the hydrogen bond with three residues, namely, Arg105 (6.03 Å), Gln110 (4.52 Å), and Ser158 (4.08 Å), and one Pi–sigma with Val104 (4.89 Å). VdW interaction with residues Phe8, Lys102, Phe103, Thr111, Asn151, Thr292, and Phe294 was also formed. In the sesamolinol molecule, one residue Ser158 (4.10 Å) formed hydrogen bond (Figure 3C). Several other residues formed other types of molecular interactions such as Ile106 (4.45 Å), Gln110 (5.21 Å) (C–H bond), Val104 (5.04 Å) (Pi–sigma), Val202 (5.45 Å), Ile249 (5.21 Å) (alkyl), His246 (5.26 Å) (Pi–alkyl), and residues Arg105, Gln107, Asn151, Asp245 demonstrated VdW interactions.

Consistent with previous studies which reported the potential inhibitors of M^pro^ (Park et al., 2015; Aanouz et al., 2020; Bello et al., 2020; Chikhale et al., 2020b; Krupanidhi et al., 2020; Matveeva et al., 2020; Muhammad et al., 2020; Tripathi et al., 2020; Mitra et al., 2021; Prasanth et al., 2021; Varadharajan et al., 2021), in our study, screened four compounds (sesamin, sesaminol, sesamolin, and sesamolinol) were found to be tightly fit into the binding pocket of the M^pro^ of COVID-19. In previous studies, the potential of herb-derived natural compounds have been explored to inhibit the M^pro^ of COVID-19 using integrated bioinformatics and molecular modeling approaches (Kumar et al., 2020; Suravajhala et al., 2020; Gunda et al., 2021; Mishra et al., 2021). Three natural metabolites, namely, ursolic acid, carvacrol, and oleanolic acid have been reported as the potential inhibitors of M^pro^ of COVID-19. The molecular docking study of ursolic acid, carvacrol, and oleanolic acid with the M^pro^ protein demonstrated the binding energy of −5.9, −4.0, and −6.0 kcal/mol, respectively (Kumar et al., 2020). The ursolic acid formed a strong hydrogen bond with Ser46 residues, while the docking study of carvacrol and oleanolic acid with the M^pro^ protein exhibits hydrogen bonding with Gly143 and Gln189 residues of the active site, respectively. In a recent study, Gunda et al. (2021) proposed the natural xanthone compounds as promising drug inhibitors against the M^pro^ of COVID-19 based on their significant antiviral power, which is well-documented in literatures. In a recent follow-up study, Mishra et al. (2021) explored a set of natural compounds to investigate their binding potential to the M^pro^ of COVID-19. Based on the docking and MD simulations, the four natural compounds, namely, amentoflavone, guggulsterone, puerarin, and piperine have been reported as antiviral compounds against the M^pro^ of COVID-19. The binding affinity of these natural metabolites with M^pro^ protein confirms the results of the present study.

Several compounds of sesame possess the natural antibacterial, antifungal, antiviral, and anti-inflammatory properties, and lignans such as sesamin, sesaminol, sesamolin, and sesamolinol are good examples (Uncu et al., 2015; Dravie et al., 2020). Sesamin is exclusively found in the sesame plant, and its antioxidant, antibacterial, antiviral, and antifungal activities are well-reported. Kodchakorn et al. (2020) identified that sesamin interacts with the M^pro^ of SARS-CoV-2 and affects the thermal stability of M^pro^ using in silico methods, providing evidence for sesamin as a structural inhibitor against the M^pro^ of SARS-CoV-2. Other studies also indicated that the sesamin compound might interact with amino acid residues Ser144, Cys145, Gln189, and Gln192 and showed significant interactions with effective residues His41, Met49, and Met165 of the M^pro^ of COVID-19 (Pandey and Verma, 2020). In a follow-up study, Allam et al. (2021) explored the sesamin and sesamolin compounds along with other natural compounds against M^pro^, PL^pro^, and RdRp proteins. Sesamin was found to be interacted with M^pro^ with three residues including Gln189, Thr190, and His41, while the sesamolin molecule was reported to interact with two amino acid residues, namely, Gln189 and Thr190. Our results may support the previous findings on the inhibitory effect of sesamin and sesamolin against the M^pro^ of COVID-19. Previous reports demonstrated the docking results only for few compounds including sesamin and sesamolin but did not consider all compounds of sesame reported in the literature, which have significant medicinal properties as well. In the present study, we explored the potential of 36 sesame-derived natural compounds against the M^pro^ of COVID-19, and based on the docking results, the four natural compounds were selected, namely, sesamin, sesaminol, sesamolin, and sesamolinol for further evaluation using MD simulations on 200 ns. The previous studies lack the evidence of docking results evaluated using MD simulations on high ns scale. The screened natural compounds based on the present study were also well-studied for their central role in different biological activities. Several *in vitro* and *in vivo* studies illustrate the neuroprotective role of sesamin against cerebral ischemia (Chung et al., 2010; Dar and Arumugam, 2013). Also, this major lignin compound has demonstrated other biological activities such as antihypertensive, atherosclerosis, thrombosis, antidiabetic, anticancer, cardiovascular, and anti-inflammatory (Kumar et al., 2018; Dalibalta et al., 2020). Of note, sesamin has been previously shown to be effective against swine flu (influenza type A H1N1) through in silico and *in vitro* studies (Fanhchaksai et al., 2015). This compound was established as a novel inhibitor of pro-inflammatory cytokines, IL-1β and TNF-α. Sesaminol is one type of sesame lignan compound commonly found in sesame seeds and well known for its strong antioxidant and anticancer properties (Miyahara et al., 2001; Watanabe et al., 2017). Using *in vitro* and *in vivo* models, Kaji et al. (2020) reported the preventive effect of sesaminol on a neurodegenerative disease named as Parkinson’s disease (PD). Sesamolin, the second major lignan, found in sesame oil has been regarded as a natural therapeutic agent because of its various therapeutic properties (Michailidis et al., 2019). Free radical scavenging activity of sesamolin provides protection to neuronal hypoxia (Park et al., 2010). Sesamolinol has also been considered the important lignin compound due to its various biological activities (Grougnet et al., 2011). The present study reported bioactive molecules (sesaminol, sesamolin, and sesamolinol) which are established as potential inhibitors of M^pro^ having enough bibliographical research support.

### Evaluation of Drug Likeness

Prior to conducting MD simulation analysis, we evaluated the pharmacokinetic properties of the screened compounds of sesame. The ADME results of the shortlisted molecules calculated using SWISSADME server are shown in Table 2. Sesamin, sesaminol, sesamolin, and sesamolinol have the following molecular weights, respectively: 354.35, 370.35, 370.35, and 372.37 g/mol; these four natural compounds have a molecular weight ≤500 g/mol, which indicated that these screened natural compounds may easily be transported, diffused, and absorbed by the body (Lipinski et al., 2001; Lipinski, 2004).The LogP values of sesamin, sesaminol, sesamolin, and sesamolinol molecules were found to have 2.79, 2.37, 2.74, and 2.56, respectively, which are in accordance with Lipinski’s rule of five. For these four compounds, the number of hydrogen bond donors was less than five, which meets the criteria of ADME as the number of H bond donors should be ≤5. The ADME analysis revealed that sesamin, sesaminol, sesamolin, and sesamolinol molecules present the following values of the topological polar surface (TPSA): 55.38, 75.61, 64.61, and 75.61 Å2. The range of lower TPSA values represents the acceptable results, as described by Ahuja et al. (2021) and Singh et al. (2021) in previous studies. It has been noted that the natural compounds derived from sesame are better behaved than the co-crystallized molecule. These screened molecules also validate Veber’s rule which state the oral bioavailability of drug-like compounds. These four metabolites, namely, sesamin, sesaminol, sesamolin, and sesamolinol have the molar refractivity values 90, 92.02, 91.52, and 93.98, respectively; these compounds also present the scores of the synthetic accessibility (SA): 4.12, 4.31, 4.43, and 4.50, respectively. SA is one of the important parameters of synthesis during the process of drug designing (Ertl and Schuffenhauer, 2009). The predicted SA score of these screened compounds was <10, which suggested that these compounds can be easily synthesized. Taken together, the drug-likeness analysis indicated that these sesame-derived natural metabolites possess favorable pharmacokinetic properties, and thus can be considered drug-like molecules.

**TABLE 2:** ADME properties of screened top four compounds from sesame (*S. indicum* L.).

Drug-likeliness properties	MW (g/mol) (range ≤500 g)	Consensus log Po/w (range ≤5)	No. of H bond acceptors (range ≤10)	No. of H bond donors (range ≤5)	Molar refractivity (range 40–130)	Lipinski	Veber	Bioavailability score (range 0.4–0.6)	Synthetic accessibility (range >6)	TPSA (Å2) (range >100)	No. of rotatable bonds (range 1–10)	Solubility (mg/ml)
Phytochemical												
Sesamin (CID_72307)	354.35	2.79	6	0	90.00	Yes	Yes	0.55	4.12	55.38	2	8.98e-03
Sesaminol (CID_94672)	370.35	2.37	7	1	92.02	Yes	Yes	0.55	4.31	75.61	2	3.62e-02
Sesamolin (CID_131801617)	370.35	2.74	7	3	91.52	Yes	Yes	0.55	4.43	64.61	3	1.75e-02
Sesamolinol (CID_443019)	372.37	2.56	7	1	93.98	Yes	Yes	0.55	4.50	75.61	4	2.80e-02

### Conformation of Stability of Docking Complexes for Natural Compounds and SARS-CoV-2 M^pro^ by Molecular Dynamics Simulations

In order to determine the structural stability of docking complexes, MD simulations were run with the most stable docked models on 200 ns. Based on docking scores, hydrogen bonds, and compound specificity, four docking complexes, namely, sesamin, sesaminol, sesamolin, and sesamolinol were subjected to MD simulations. High binding energy scores of docking complexes allowed for the estimation of the amino acid residue interactions over time. The RMSD, RMSF, SASA, and Rg plots were calculated to evaluate the stability of simulated systems.

### Root-Mean-Square Deviation

**FIGURE 4:**
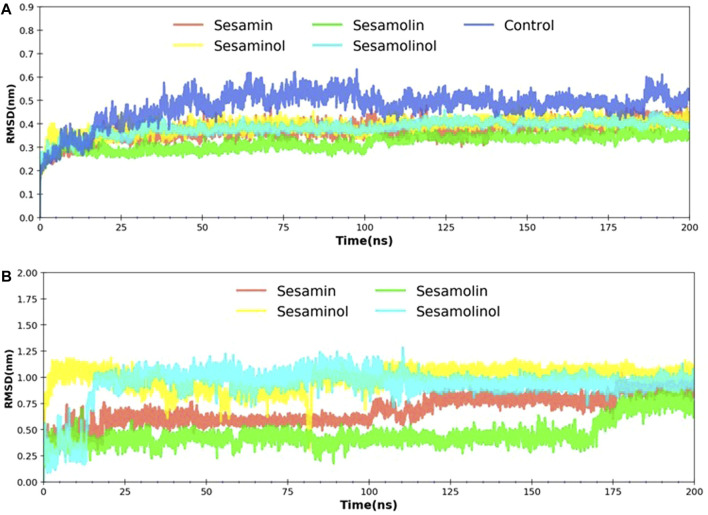
RMSD analysis. **(A)** Backbone RMSD plot of docking complexes; and **(B)** ligand RMSD plot of complexes [M^pro^ – sesamin complex (red), M^pro^ – sesamolin complex (green), M^pro^ – sesaminol complex (yellow), M^pro^ – sesamolinol complex (cyan), and control (blue)].

The RMSD is a most commonly used quantitative method to evaluate the stability of the docking complexes and measures the conformational stability perturbations within the protein backbone during MD simulations on different nanosecond scales (Sargsyan et al., 2017). In order to investigate the stability of the ligand molecules to the protein, all the ligand and backbone RMSDs were graphically measured. As evident from Figure 4A, the protein backbone of M^pro^ showed constant stability throughout the simulation with a range between 0.37 and 0.47 nm. The average RMSD values for complexes with sesamin, sesaminol, sesamolin, and sesamolinol were ∼0.37, ∼ 0.38, ∼ 0.31, and ∼0.38 nm, respectively. Likewise, the control (blue) element also showed the average value of RMSD to be around 0.47 nm. The complex with sesaminol (yellow) and sesamolinol (cyan) displayed higher simulation trajectory after ∼50 ns than the complex with sesamin (red) and sesamolin (green). The compound sesamolin has shown two fluctuations throughout the simulations on 200 ns time scale. The first stable conformation was noted between 25 and 100 ns, and the second stable conformation was found between 110 and 200 ns. The RMSD constant was at ∼0.25, and a large fluctuation was observed between 10 and 25 ns. However, there was no significant effect of this fluctuation was found on the protein structure. Sesamolinol showed slight changes in the starting period of simulation between 2 and 25 ns. After 25 ns, sesamolinol was found to be constant at ∼0.35 throughout the simulations. It may be because of the binding region size and loop presence at the pocket site. All the four ligand molecules shared the almost similar trend of stability and RMSD values with small conformational changes. As depicted in Figure 4B, the calculated ligand RMSD plot is the conformation of the measured protein backbone; RMSD plot shows the stability of target compounds throughout the simulation with fluctuation in sesamolinol at starting point between 5 and 15 ns on ∼0.50 nm. In the same plot, the sesamolin compound also showed the fluctuation between 160 and 170 ns on ∼0.25 nm. Based on the minimal fluctuations and low difference in values depicted in the protein backbone and ligand RMSD plots, it can be predicted that protein–ligand complexes were stable and comparable to solved structures. The docked pose of our ligands is fixed in the active region, same as the crystal structure ligand Z45617795, which is quite acceptable in protein–ligand interaction (Table 1). The RMSDs of our ligands with heavy atoms are similar to crystal structure resolution which is higher than 1.65 Å and is accurately ordered and exactly fitted in the electron density map. Therefore, RMSDs obtained from MD simulation also showed the structure stability during simulation (each ligand has remained constant and has a constant range of RMSDs). From these observations, we assume that our ligand RMSDs (0.25–1 nm) showed similar stability as crystal ligand pose has in resolutions.

### Root-Mean-Square Fluctuation

**FIGURE 5:**
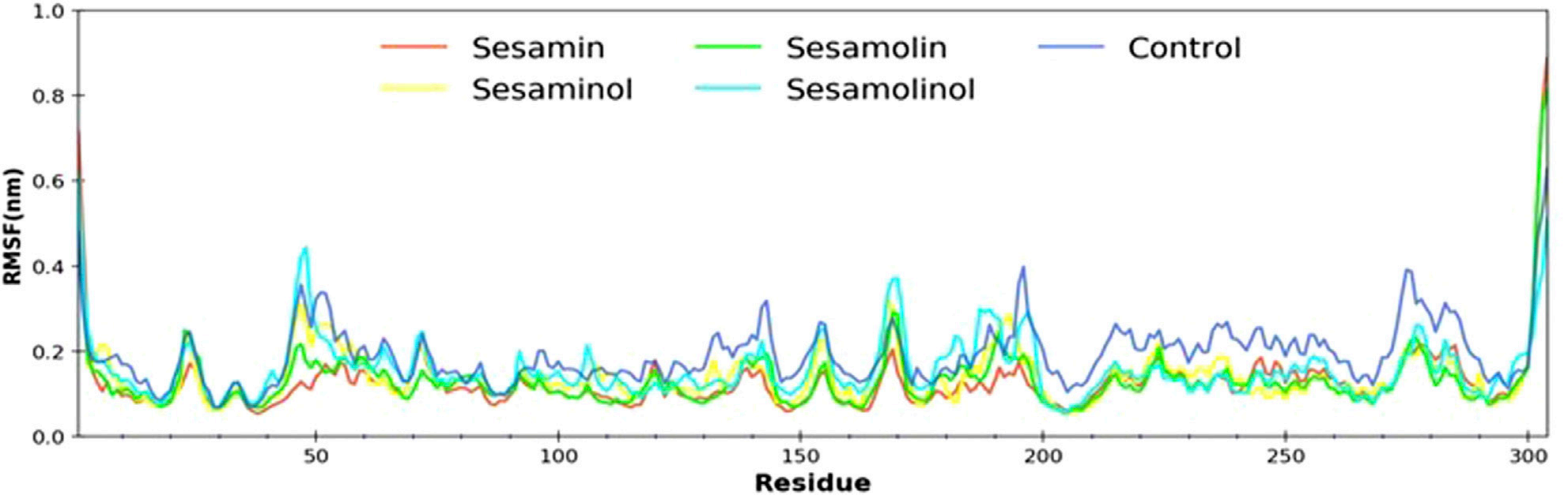
Calculated RMSF plot of docking complexes[M^pro^ – sesamin complex (red), M^pro^ – sesamolin complex (green), M^pro^ – sesaminol complex (yellow), M^pro^ – sesamolinol complex (cyan), and control (blue)].

In order to determine the individual residue flexibility of the system with the time, RMSF was calculated, in which high fluctuation score indicates more flexibility and unstable bonds, while a low score reflects well-structured regions in the protein–ligand complexes (Gajula et al., 2016). The RMSF of alpha-carbon atoms of all system was investigated and is given in Figure 5. All the five systems (control, M^pro^–sesamin, M^pro^–sesaminol, M^pro^–sesamolin, and M^pro^–sesamolinol complexes) demonstrated almost a similar pattern of fluctuation across the whole structure during simulation. The average RMSF values of control, M^pro^– sesamin, M^pro^– sesaminol, M^pro^– sesamolin, and M^pro^– sesamolinol complexes were ∼0.25, ∼0.20, ∼0.23, ∼0.21, and ∼0.35 nm, respectively. These values revealed that all the subjected docking complexes exhibit relatively less conformation fluctuation than the control system. These less fluctuations of the docking complexes suggested that the residues distributed across the active site of M^pro^ interact with sesamin, sesaminol, sesamolin, and sesamolinol in a significant manner.

### Hydrogen Bond Analysis

**FIGURE 6:**
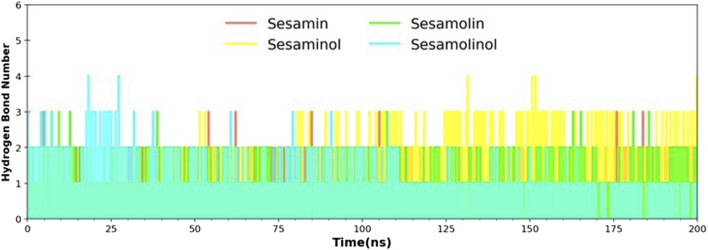
Hydrogen bond analysis of docking complexes [M^pro^ – sesamin complex (red), M^pro^ – sesamolin complex (green), M^pro^ – sesaminol complex (yellow), and M^pro^ – sesamolinol complex (cyan)].

Hydrogen bonds play an essential role in establishing molecular interactions of biological systems. The molecular interaction between M^pro^ and sesame-derived bioactive molecules was explored by the secondary structure changes, which is, in turn, regulated by a number of hydrogen bonds. For selected complexes (M^pro^– sesamin, M^pro^– sesaminol, M^pro^– sesamolin, and M^pro^– sesamolinol), a number of formed hydrogen bonds were calculated throughout the MD simulation on the scale of 200 ns. The number of hydrogen bonds and hydrogen bond distribution is represented in Figure 6. In complex with sesaminol (yellow) and sesamolinol (cyan), the numbers of hydrogen bonds were three, with few conformations showing up to 4 hydrogen bonds throughout the simulations. Sesamin (red) and sesamolin (green) have a constant range of hydrogen bonds between two and three in whole simulation. These results showed that the screened natural metabolites were able to maintain a strong interaction with a pocket site and suggested that all four docking complexes were stable throughout the simulation.

### Radius of Gyration, and Solvent Accessible Surface Area Analysis

**FIGURE 7:**
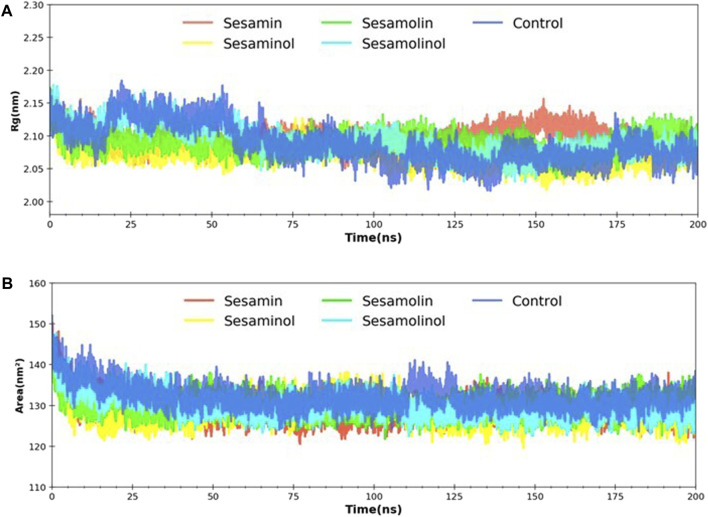
Rg and SASA analysis. **(A)** Predicted Rg plot of docking complexes; and **(B)** SASA plot of selected complexes [M^pro^ – sesamin complex (red), M^pro^ – sesamolin complex (green), M^pro^ – sesaminol complex (yellow), M^pro^ – sesamolinol complex (cyan), and control (blue)].

MD trajectories corresponding to four complexes (M^pro^– sesamin, M^pro^– sesaminol, M^pro^– sesamolin, and M^pro^– sesamolinol) were further investigated with the aid of Rg and SASA analysis. Rg was calculated with a primary goal to determine the compactness of the system with the time. As depicted in Figure 7A, the Rg values of all four systems with control were reported as 2.08–2.15 nm throughout the simulation. Rg value analysis affirms the stability of each system and suggested that the binding of screened natural phytochemicals does not induce structural changes during whole simulation. During simulation, SASA values were calculated to measure the receptor exposed to the solvents. It is well-documented that a higher SASA value reflects the expansion of protein volume during MD simulation (Kumar et al., 2020). Always, a low fluctuation is expected during whole simulation. Interaction with ligand compounds may influence SASA and sometimes affect the protein structure in a significant manner. The calculated SASA values showed between 130 and 148 nm^2^, reflecting that the binding of sesamin, sesaminol, sesamolin, and sesamolinol does not affect the folding of protein (Figure 7B). The calculated SASA values for these ligand compounds are the consent of previous reports (Kumar et al., 2020; Mishra et al., 2021) and suggested that all of the four complexes were stable after the binding of sesamin, sesaminol, sesamolin, and sesamolinol to the M^pro^ active site.

### Estimation of Binding Free Energy

The average free binding energy of selected complexes (M^pro^– sesamin, M^pro^– sesaminol, M^pro^– sesamolin, and M^pro^– sesamolinol) was calculated by using a python script MmPbSaStat.py embedded in g_mmpbsa package. The molecular mechanic/Poisson–Boltzmann surface area (MM/PBSA) is one of the popular and accurate methods to estimate the ligand binding affinities in the protein system. To calculate the binding free energy, we have utilized the steps previously described (Gajula et al., 2016; Jee et al., 2017). The MM/PBSA-based binding energy score extracted after the systematical calculation is provided in Table 3. The cumulative sum of different energies such as van der Walls, electrostatic, polar solvation, and SASA is presented as the final binding energy. All types of the energy significantly contributed to the molecular interaction between the ligand compounds and M^pro^. The evaluated binding free energy of screened molecules exhibited as sesamin (−145.511 ± 17.054 kJ/mol), sesamolin (−211.240 ± 14.034 kJ/mol), sesaminol (−149.078 ± 9.043 kJ/mol) and sesamolinol (−199.110 ± 15.881 kJ/mol). The negative values of the binding energy reflect that the targeted compound favorably interact with the receptor protein. As compared with other screened compounds, the sesamolin (−211.240 ± 14.034 kJ/mol) showed the maximum negative binding energy. The MM/PBSA results clearly suggest that sesamolinol (−199.110 ± 15.881 kJ/mol) possessed the second least binding energy. These natural compounds with the maximum negative binding energy and better binding affinity could be utilized as potential inhibitors against the M^pro^ of COVID-19.

**TABLE 3:** Calculated total binding energy, van der Waals energy, electrostatic energy, polar solvation energy, and SASA energy of the docking complexes.

Complex	Binding energy (kJ/mol)	van der Waals energy (∆EvdW) (kJ/mol)	Electrostatic energy (∆Elec), (kJ/mol)	Polar solvation energy (∆G polar) (kJ/mol)	SASA energy (kJ/mol)
Sesamin	−145.511 ± 17.054	−185.239 ± 12.497	−1.331 ± 2.720	56.328 ± 10.084	−15.269 ± 0.859
Sesamolin	−211.240 ± 14.034	−244.688 ± 13.232	−2.394 ± 2.452	53.429 ± 6.865	−17.587 ± 0.839
Sesaminol	−149.078 ± 9.043	−158.179 ± 8.593	−1.087 ± 1.785	24.598 ± 4.487	−14.410 ± 0.870
Sesamolinol	−199.110 ± 15.881	−233.811 ± 13.828	2.162 ± 2.619	51.381 ± 7.632	−18.842 ± 0.954

## Conclusion

The inhibition of M^pro^ protein represents a promising strategy for controlling viral replication leading to discovery of potential drug candidates. The current extensive study concludes four phytochemicals, namely, sesamin, sesaminol, sesamolin, and sesamolinol as potential inhibitors against the M^pro^ of SARS-CoV-2. The integrated molecular docking and MD simulation study revealed that these bioactive molecules form a very stable complex with M^pro^ that shows excellent binding affinities higher than other sesame-derived molecules. Docking complexes of these natural metabolites with M^pro^ showed a stable conformation on 200 ns, which is further supported by the results of binding free energy. Moreover, the proposed potential inhibitors also meet the criteria of drug likeness based on Lipinski’s rule of five and ADME properties. The inhibitory effect of these sesame-derived natural compounds against the M^pro^ of SARS-CoV-2 may also be further validated using a plethora of *in vitro* and *in vivo* experiments. The current study suggested that the screened phytochemicals (sesamin, sesaminol, sesamolin, and sesamolinol) have shown enough potential to inhibit the M^pro^ and may be utilized as effective drug candidates for the development of new treatment against COVID-19 infection.

## Data Availability

The datasets presented in this study can be found in online repositories. The names of the repository/repositories and accession number(s) can be found in the article/Supplementary Material.
