# Supplementary material for: Inhibition Potencies of Phytochemicals Derived from Sesame Against SARS-CoV-2 Main Protease: A Molecular Docking and Simulation Study
Source: Front Chem. 2021 Oct 8;9:744376. doi: 10.3389/fchem.2021.744376 (PMC8531729; doi:10.3389/fchem.2021.744376)
Supplement: Supplementary file 1 [file Table1.DOCX]

Supplementary Material

**Supplementary Table 1.** List of natural compounds exclusively found in Sesame (*Sesamum indicum* L.)**.**

| **S.No** | **Compound Name** | **PubChem ID** | **Molecular Formula** | **Molecular**  **Weight (g/mol)** | **Chemical Structure** | |
| --- | --- | --- | --- | --- | --- | --- |
|  |  |  |  |  | **2D** | **3D** |
| 1. | Latifonin | [CID_156633](https://pubchem.ncbi.nlm.nih.gov/compound/156633) | [C_11_H_16_O_7_](https://pubchem.ncbi.nlm.nih.gov/#query=C11H16O7) | 260.24 | 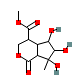 | 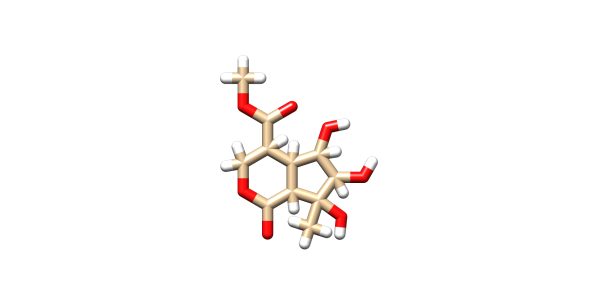 |
| 2. | Momor-Cerebroside I | CID_[91895300](https://pubchem.ncbi.nlm.nih.gov/compound/91895300) | [C_48_H_93_NO_10_](https://pubchem.ncbi.nlm.nih.gov/#query=C48H93NO10) | 844.3 | 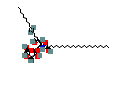 | 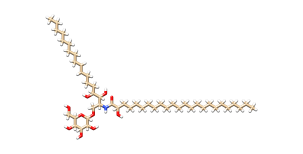 |
| 3. | Soyacerebroside II | [CID_6450042](https://pubchem.ncbi.nlm.nih.gov/compound/6450042) | [C_40_H_75_NO_9_](https://pubchem.ncbi.nlm.nih.gov/#query=C40H75NO9) | 714 | 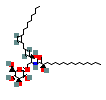 | 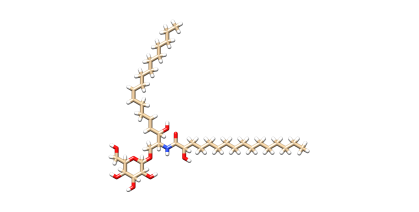 |
| 4. | Aurantiamide acetate | CID_[9832120](https://pubchem.ncbi.nlm.nih.gov/compound/9832120) | [C_27_H_28_N_2_O_4_](https://pubchem.ncbi.nlm.nih.gov/#query=C27H28N2O4) | 444.5 | 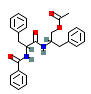 | 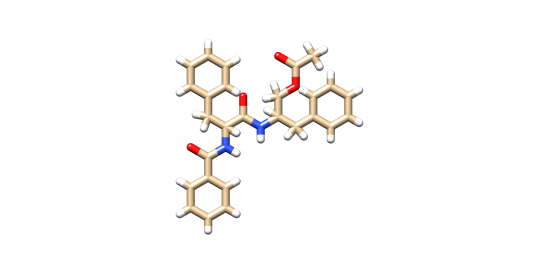 |
| 5. | Beta-sitosterol | CID_ [222284](https://pubchem.ncbi.nlm.nih.gov/compound/222284) | [C_29_H_50_O](https://pubchem.ncbi.nlm.nih.gov/#query=C29H50O) | 414.7 | 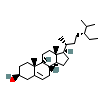 | 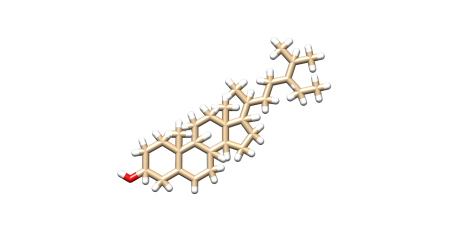 |
| 6. | Daucosterol | CID_[5742590](https://pubchem.ncbi.nlm.nih.gov/compound/5742590) | [C_35_H_60_O_6_](https://pubchem.ncbi.nlm.nih.gov/#query=C35H60O6) | 576.8 | 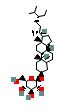 | 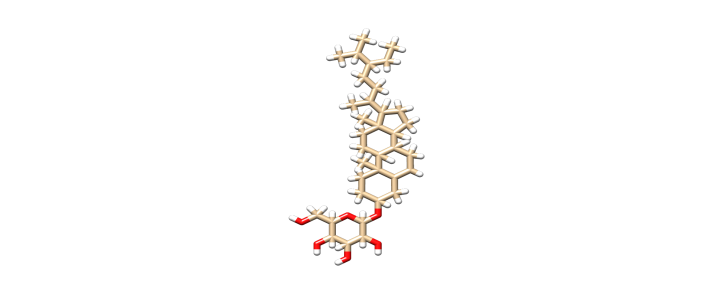 |
| 7. | D-Galactitol | CID_ [11850](https://pubchem.ncbi.nlm.nih.gov/compound/Galactitol) | [C_6_H_14_O_6_](https://pubchem.ncbi.nlm.nih.gov/#query=C6H14O6) | 182.17 | 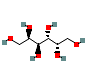 | 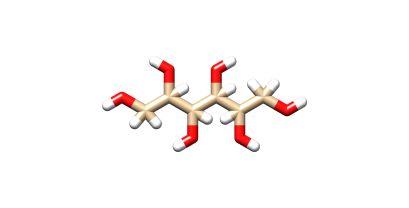 |
| 8. | Sesamolin | CID_[131801617](https://pubchem.ncbi.nlm.nih.gov/compound/131801617) | [C_20_H_18_O_7_](https://pubchem.ncbi.nlm.nih.gov/#query=C20H18O7) | 370.4 | 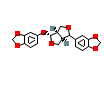 | 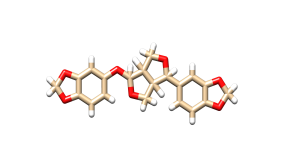 |
| 9. | Dronabinol | CID_ [16078](https://pubchem.ncbi.nlm.nih.gov/compound/16078) | [C_21_H_30_O_2_](https://pubchem.ncbi.nlm.nih.gov/#query=C21H30O2) | 314.5 | 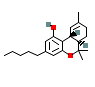 | 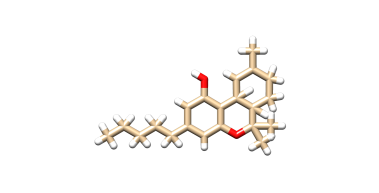 |
| 10. | 1,2-Dilinoleoyl-3-Palmitoyl-Rac-Glycerol | CID_[9544106](https://pubchem.ncbi.nlm.nih.gov/compound/9544106) | [C_55_H_98_O_6_](https://pubchem.ncbi.nlm.nih.gov/#query=C55H98O6) | 855.4 | 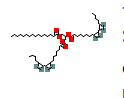 | 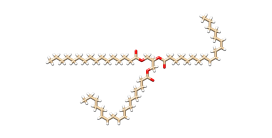 |
| 11. | 1,2-Dilinoleoyl-3-Oleoyl-Rac-Glycerol | CID_ [9544291](https://pubchem.ncbi.nlm.nih.gov/compound/9544291) | [C_57_H_100_O_6_](https://pubchem.ncbi.nlm.nih.gov/#query=C57H100O6) | 881.4 | 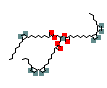 | 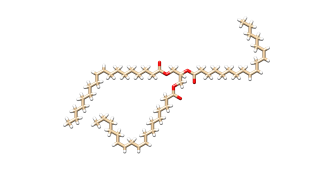 |
| 12. | Linoleic Acid | CID_  [5280450](https://pubchem.ncbi.nlm.nih.gov/compound/5280450) | [C_18_H_32_O_2_](https://pubchem.ncbi.nlm.nih.gov/#query=C18H32O2) | 280.4 | 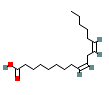 | 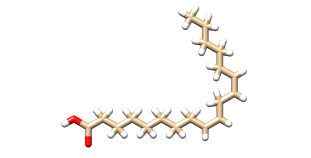 |
| 13. | Linolenic Acid | CID_[5280934](https://pubchem.ncbi.nlm.nih.gov/compound/5280934) | [C_18_H_30_O_2_](https://pubchem.ncbi.nlm.nih.gov/#query=C18H30O2) | 278.4 | 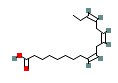 | 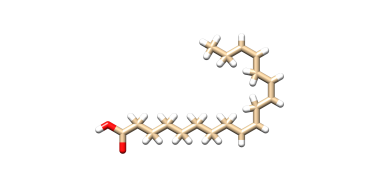 |
| 14. | Oleic Acid | CID_  [445639](https://pubchem.ncbi.nlm.nih.gov/compound/445639) | [C_18_H_34_O_2_](https://pubchem.ncbi.nlm.nih.gov/#query=C18H34O2) | 282.5 | 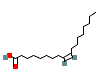 | 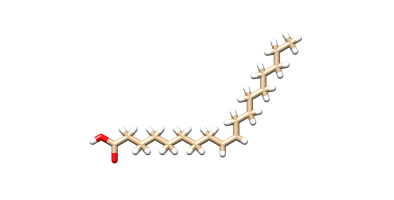 |
| 15. | Palmitic Acid | CID_ [985](https://pubchem.ncbi.nlm.nih.gov/compound/985) | [C_16_H_32_O_2_](https://pubchem.ncbi.nlm.nih.gov/#query=C16H32O2) | 256.42 | 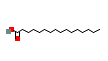 | 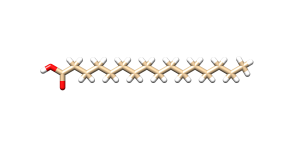 |
| 16. | Pinoresinol | CID_  [73399](https://pubchem.ncbi.nlm.nih.gov/compound/73399) | [C_20_H_22_O_6_](https://pubchem.ncbi.nlm.nih.gov/#query=C20H22O6) | 358.4 | 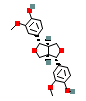 | 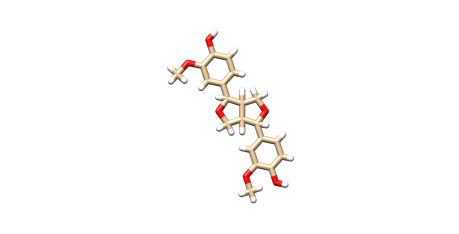 |
| 17. | Sesamin | CID_ [72307](https://pubchem.ncbi.nlm.nih.gov/compound/72307) | [C_20_H_18_O_6_](https://pubchem.ncbi.nlm.nih.gov/#query=C20H18O6) | 354.4 | 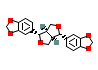 | 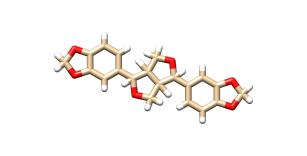 |
| 18. | Sesaminol | CID_ [94672](https://pubchem.ncbi.nlm.nih.gov/compound/94672) | [C_20_H_18_O_7_](https://pubchem.ncbi.nlm.nih.gov/#query=C20H18O7) | 370.4 | 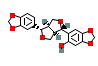 | 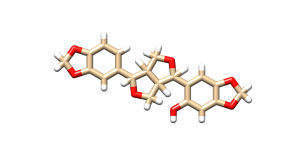 |
| 19. | Sesamol | CID_ [68289](https://pubchem.ncbi.nlm.nih.gov/compound/68289) | [C_7_H_6_O_3_](https://pubchem.ncbi.nlm.nih.gov/#query=C7H6O3) | 138.12 | 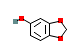 | 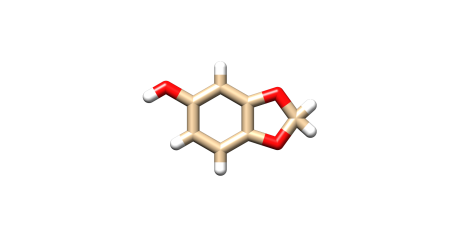 |
| 20. | Sesamolinol | CID_ [443019](https://pubchem.ncbi.nlm.nih.gov/compound/443019) | [C_20_H_20_O_7_](https://pubchem.ncbi.nlm.nih.gov/#query=C20H20O7) | 372.4 | 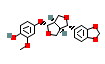 | 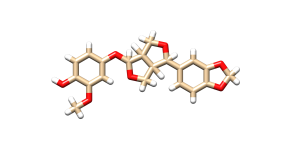 |
| 21. | Stearic Acid | CID_ [5281](https://pubchem.ncbi.nlm.nih.gov/compound/5281) | [C_18_H_36_O_2_](https://pubchem.ncbi.nlm.nih.gov/#query=C18H36O2) | 284.5 | 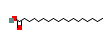 | 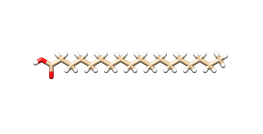 |
| 22. | Tocopherol | CID_ [14985](https://pubchem.ncbi.nlm.nih.gov/compound/14985) | [C_29_H_50_O_2_](https://pubchem.ncbi.nlm.nih.gov/#query=C29H50O2) | 430.7 | 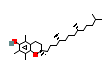 | 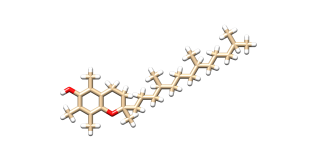 |
| 23. | Vitamin A Retinol | CID_ [445354](https://pubchem.ncbi.nlm.nih.gov/compound/445354) | [C_20_H_30_O](https://pubchem.ncbi.nlm.nih.gov/#query=C20H30O) | 286.5 | 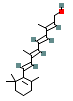 | 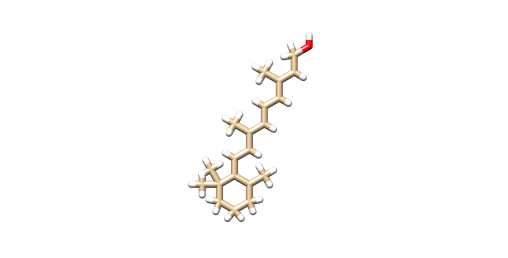 |
| 24. | Vitamin K Phylloquinone | CID_ [5284607](https://pubchem.ncbi.nlm.nih.gov/compound/5284607) | [C_31_H_46_O_2_](https://pubchem.ncbi.nlm.nih.gov/#query=C31H46O2) | 450.7 | 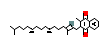 | 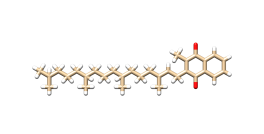 |
| 25. | Arachidic acid | CID_ [10467](https://pubchem.ncbi.nlm.nih.gov/compound/10467) | [C_20_H_40_O_2_](https://pubchem.ncbi.nlm.nih.gov/#query=C20H40O2) | 312.5 | 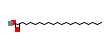 | 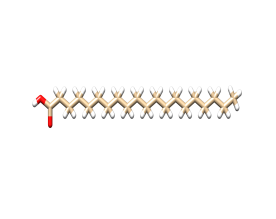 |
| 26. | Myristic acid | CID_ [11005](https://pubchem.ncbi.nlm.nih.gov/compound/11005) | [C_14_H_28_O_2_](https://pubchem.ncbi.nlm.nih.gov/#query=C14H28O2) | 228.37 | 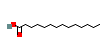 | 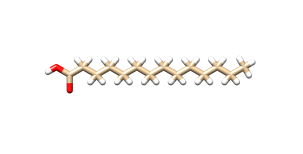 |
| 27. | Behenic acid | CID_ [8215](https://pubchem.ncbi.nlm.nih.gov/compound/8215) | [C_22_H_44_O_2_](https://pubchem.ncbi.nlm.nih.gov/#query=C22H44O2) | 340.6 | 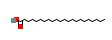 | 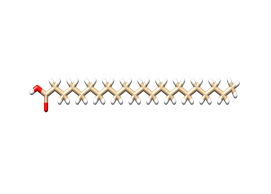 |
| 28. | Octanoic acid | CID_ [379](https://pubchem.ncbi.nlm.nih.gov/compound/379) | [C_8_H_16_O_2_](https://pubchem.ncbi.nlm.nih.gov/#query=C8H16O2) | 144.21 | 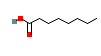 | 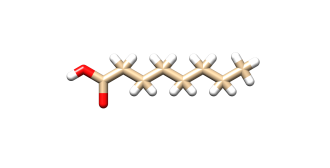 |
| 29. | Decanoic acid | CID_ [2969](https://pubchem.ncbi.nlm.nih.gov/compound/2969) | [C_10_H_20_O_2_](https://pubchem.ncbi.nlm.nih.gov/#query=C10H20O2) | 172.26 | 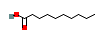 | 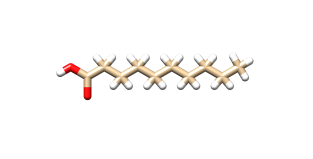 |
| 30. | Lauric acid | CID_ [3893](https://pubchem.ncbi.nlm.nih.gov/compound/3893) | [C_12_H_24_O_2_](https://pubchem.ncbi.nlm.nih.gov/#query=C12H24O2) | 200.32 | 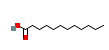 | 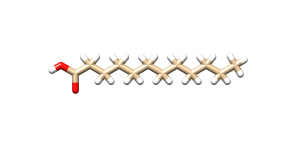 |
| 31. | Eicosenoic acid | CID_ [5282768](https://pubchem.ncbi.nlm.nih.gov/compound/5282768) | [C_20_H_38_O_2_](https://pubchem.ncbi.nlm.nih.gov/#query=C20H38O2) | 310.5 | 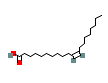 | 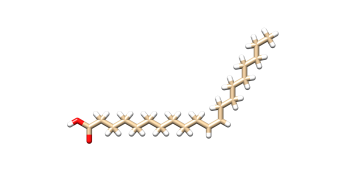 |
| 32. | Lariciresinol | CID_ [332427](https://pubchem.ncbi.nlm.nih.gov/compound/332427) | [C_20_H_24_O_6_](https://pubchem.ncbi.nlm.nih.gov/#query=C20H24O6) | 360.4 | 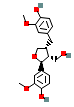 | 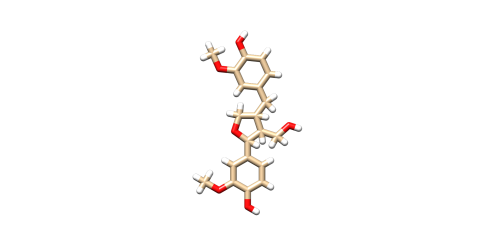 |
| 33. | Campesterol | CID_ [173183](https://pubchem.ncbi.nlm.nih.gov/compound/173183) | [C_28_H_48_O](https://pubchem.ncbi.nlm.nih.gov/#query=C28H48O) | 400.7 | 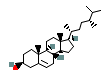 | 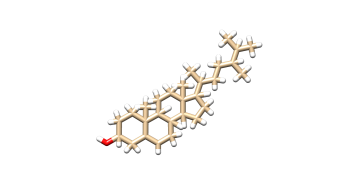 |
| 34. | Δ5‐Avenasterol | CID_[5281326](https://pubchem.ncbi.nlm.nih.gov/compound/5281326) | [C_29_H_48_O](https://pubchem.ncbi.nlm.nih.gov/#query=C29H48O) | 412.7 | 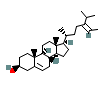 | 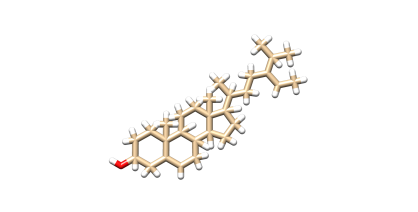 |
| 35. | Δ7‐Stigmasterol | CID_[131751494](https://pubchem.ncbi.nlm.nih.gov/compound/131751494) | [C_29_H_46_O](https://pubchem.ncbi.nlm.nih.gov/#query=C29H46O) | 410.7 | 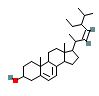 | 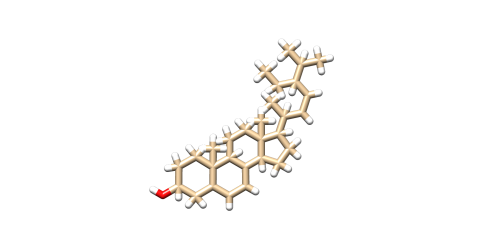 |
| 36. | Δ7‐Avenasterol | CID_[12795736](https://pubchem.ncbi.nlm.nih.gov/compound/12795736) | [C_29_H_48_O](https://pubchem.ncbi.nlm.nih.gov/#query=C29H48O) | 412.7 | 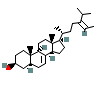 | 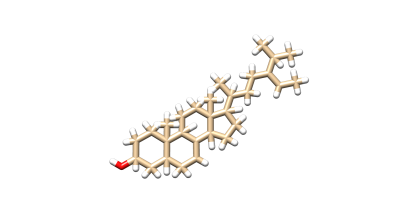 |
